# Chronic Low-Dose Exposure to Xenoestrogen Ambient Air Pollutants and Breast Cancer Risk: XENAIR Protocol for a Case-Control Study Nested Within the French E3N Cohort

**DOI:** 10.2196/15167

**Published:** 2020-09-15

**Authors:** Amina Amadou, Thomas Coudon, Delphine Praud, Pietro Salizzoni, Karen Leffondre, Emilie Lévêque, Marie-Christine Boutron-Ruault, Aurélie M N Danjou, Xavier Morelli, Charlotte Le Cornet, Lionel Perrier, Florian Couvidat, Bertrand Bessagnet, Julien Caudeville, Elodie Faure, Francesca Romana Mancini, John Gulliver, Gianluca Severi, Béatrice Fervers

**Affiliations:** 1 Department of Prevention Cancer Environment Centre Léon Bérard Lyon France; 2 Inserm UA 08 Radiations: Défense, Santé, Environnement Lyon France; 3 Ecole Centrale de Lyon INSA Université Claude Bernard Lyon 1 Ecully France; 4 ISPED, Inserm U1219 Bordeaux Population Health Center Université de Bordeaux Bordeaux France; 5 Centre de Recherche en Epidémiologie et Santé des Populations (CESP, Inserm U1018) Faculté de Médecine Université Paris-Saclay Villejuif France; 6 Division of Cancer Epidemiology German Cancer Research Center (DKFZ) Heidelberg Germany; 7 Univ Lyon Centre Léon Bérard GATE L-SE UMR 5824 Lyon France; 8 National Institute for industrial Environment and Risks (INERIS) Verneuil-en-Halatte France; 9 Centre for Environmental Health and Sustainability School of Geography, Geology and the Environment University of Leicester Leicester United Kingdom

**Keywords:** breast cancer, hormone receptor status, air pollution, endocrine disruptors, multipollutant, geographic information system, land use regression, chemistry-transport model, epigenetic, gene-environment interaction, prospective study

## Abstract

**Background:**

Breast cancer is the most frequent cancer in women in industrialized countries. Lifestyle and environmental factors, particularly endocrine-disrupting pollutants, have been suggested to play a role in breast cancer risk. Current epidemiological studies, although not fully consistent, suggest a positive association of breast cancer risk with exposure to several International Agency for Research on Cancer Group 1 air-pollutant carcinogens, such as particulate matter, polychlorinated biphenyls (PCB), dioxins, Benzo[a]pyrene (BaP), and cadmium. However, epidemiological studies remain scarce and inconsistent. It has been proposed that the menopausal status could modify the relationship between pollutants and breast cancer and that the association varies with hormone receptor status.

**Objective:**

The XENAIR project will investigate the association of breast cancer risk (overall and by hormone receptor status) with chronic exposure to selected air pollutants, including particulate matter, nitrogen dioxide (NO2), ozone (O3), BaP, dioxins, PCB-153, and cadmium.

**Methods:**

Our research is based on a case-control study nested within the French national E3N cohort of 5222 invasive breast cancer cases identified during follow-up from 1990 to 2011, and 5222 matched controls. A questionnaire was sent to all participants to collect their lifetime residential addresses and information on indoor pollution. We will assess these exposures using complementary models of land-use regression, atmospheric dispersion, and regional chemistry-transport (CHIMERE) models, via a Geographic Information System. Associations with breast cancer risk will be modeled using conditional logistic regression models. We will also study the impact of exposure on DNA methylation and interactions with genetic polymorphisms. Appropriate statistical methods, including Bayesian modeling, principal component analysis, and cluster analysis, will be used to assess the impact of multipollutant exposure. The fraction of breast cancer cases attributable to air pollution will be estimated.

**Results:**

The XENAIR project will contribute to current knowledge on the health effects of air pollution and identify and understand environmental modifiable risk factors related to breast cancer risk.

**Conclusions:**

The results will provide relevant evidence to governments and policy-makers to improve effective public health prevention strategies on air pollution. The XENAIR dataset can be used in future efforts to study the effects of exposure to air pollution associated with other chronic conditions.

**International Registered Report Identifier (IRRID):**

DERR1-10.2196/15167

## Introduction

### Background

Breast cancer is the most common cancer among women worldwide, with an estimated 2.09 million new cancer cases diagnosed globally in 2018 [1]. Over the past 30 years, its incidence has continuously increased in France [1,2]. The rapid increase in the worldwide incidence of breast cancer has been associated with mass screening, menopausal hormonal therapy, and societal changes impacting individuals’ lifestyles. Epidemiological studies have suggested an important role of lifestyle and environmental factors, supported by geographical variations of breast cancer incidence and time trends in incidence rates among migrant populations [3-5]. However, a considerable proportion of the risk remains unexplained, and the impact of environmental factors in the etiology of breast cancer has not been fully explored. Epidemiology and laboratory findings suggest that exposure to environmental pollutants, particularly those with estrogenic potential, may influence the development of breast cancer [6,7].

Ambient air pollution is a major public health concern related to a range of adverse health effects, including cancer, and accounting for an estimated 4.2 million deaths per year [8]. In 2013, the International Agency for Research on Cancer (IARC) classified outdoor air pollution as a whole and particulate matter as carcinogenic in humans, principally based on studies of lung and bladder cancers [9]. However, studies on breast cancer are scarce, and results remain inconsistent [10]. A recent meta-analysis of individual data from 15 European cohorts revealed no association between postmenopausal breast cancer and exposure to various classes of particulate matter (PM_2.5_, PM_10_, and PM coarse); however, they reported a statistically significant positive relationship per 20 µg/m^3^ increase in nitrogen dioxide (NO_2_) exposure [11]. Epidemiological evidence suggests an association between breast cancer and NO_2_ from traffic-related air pollution [12-15]. Furthermore, women with extremely dense mammography density, a well-established risk factor for breast cancer, were less likely to have higher levels of ozone (O_3_) exposure [16]. Breast cancer has also been linked to exposure to endocrine-disrupting pollutants, such as polychlorinated biphenyls (PCBs) [17,18], dioxins [19-21], benzo[a]pyrene (BaP) [12,22], and cadmium [23,24] with sometimes diverging results. Furthermore, studies have reported positive associations between traffic-related BaP exposure as a surrogate for Polycyclic aromatic hydrocarbon (PAH) exposure and breast cancer [25,26]. Cadmium has been classified by IARC as carcinogenic to humans (Group 1), with sufficient evidence for lung cancer [27]. The only study performed on airborne cadmium exposure and breast cancer risk found no evidence of overall increased risk; however, elevated risks for hormone receptor-negative tumors (estrogen receptor and progesterone receptor-negative (ER-PR–)) were observed with higher exposure to cadmium [28].

Indoor pollution is also an important challenge for global health. Around 3 billion people use traditional biomass fuels for household cooking and heating [29], which is a major source of indoor air pollution and exposure to dioxins, PAH, particulate matter, O_3_, and NO_2_ [30-35]. However, only two studies have investigated the impact of indoor pollution on breast cancer risk [36,37]. Overall, current epidemiological evidence regarding the impact of exposure to ambient air pollution and indoor pollution on breast cancer risk is not fully consistent. In addition, the effects of simultaneous or sequential exposures to multiple compounds have been insufficiently explored, and the evidence supporting the differential effects of menopausal status on breast cancer risk and receptor status is also limited [23]. Breast cancer is no longer considered a homogeneous disease but is a heterogeneous disease composed of several distinct molecular subtypes according to hormone receptor status (ER, PR, and human epidermal growth factor receptor 2, or HER2) [38]. These subtypes each have different prognoses and can affect women differently. Molecular pathological epidemiology, which integrates molecular pathology into epidemiological studies has emerged in order to test for a difference between the association of a specific environmental exposure with subtypes classified by molecular features in determining disease incidence/mortality [39,40].

One of the major limitations of previous studies is the lack of past residential history and/or historical ambient air pollution exposure estimates, which may have resulted in exposure misclassification, contributing to imprecise risk estimates and bias towards the null [41]. Many epidemiological studies have relied on a variety of exposure assessment techniques such as using data from centrally located ambient air quality monitoring networks, surrogates for exposures. However, such methods are often insufficient for capturing the spatial variability of pollutant concentrations at the local scale, both at the intra-urban scale, and the suburban and rural scale [42]. More complex techniques have been used in recent studies to adequately represent the spatial-temporal variation of pollutants, including land-use regression models, dispersion modeling, chemistry transport models, and hybrid models [43]. These complex methods can be combined to assess exposure to each pollutant at a fine spatial scale and over a large area between 1990 and 2011.

### Life-Course Trajectories

Exposures occurring early in life and/or during biological windows of greater sensitivity (ie, in utero and during childhood) have been suggested to be more strongly associated with breast cancer risk [44,45]. However, very few studies have investigated these effects, and the majority of research has been based on adulthood exposures within short observation periods. These studies may have missed critical windows or the cumulative effects of lifetime exposure that could impact breast cancer risk [45,46]. A lack of historical measures makes retrospective exposure reconstruction difficult, especially for earlier periods. Higher breast cancer incidence is commonly observed in large cities compared with rural areas [47]. Also, women born in an urban area are at a greater risk of breast cancer versus women born in rural areas [47]. To our knowledge, no study has investigated the effects of life-course residential trajectories on breast cancer. Residence in an urban area has been suggested to be a surrogate of air pollution exposure released from road traffic, industrial facilities, and waste incineration [48], and to be useful to investigate earlier periods where historical air pollution records are unavailable and back-extrapolation unfeasible [47].

### Cumulative and Multiple Exposures

Early evidence suggests that cancer risk may not be a linear function of cumulative carcinogen dose [49]. Individuals are exposed simultaneously to a complex and changing mixture of environmental exposures [50]. These may independently, cumulatively, or interactively influence the risk of developing breast cancer. Furthermore, in single pollutant models, it is unclear whether an observed association is due to the effect of the evaluated pollutant or whether it acts as a surrogate for another pollutant from the same source. Multipollutant approaches need to address the complex structure of mixtures that frequently present multicollinearity. However, such epidemiological studies are limited [51,52].

### Gene × Environment Interactions

Current evidence on the role of genetic susceptibility (polymorphisms) related to exposure to ambient air pollution remains limited. Previous epidemiological studies have reported interactions between genetic polymorphisms and some pollutants [53,54]. Saintot et al reported that women carrying the Val CYP1B1 allele and who had lived near a waste incinerator for more than 10 years had a greater risk of breast cancer than those with the Leu/Leu genotype and had never been exposed [55]. A positive association was found in postmenopausal women with a CYP1A1 variant genotype [56].

### DNA Methylation

Methylation is one of several epigenetic events involved in the regulation of gene expression, and it can undergo alterations as a consequence of environmental stimuli. Emerging evidence suggests that exposure to ambient air pollutions could influence DNA methylation, producing hypomethylation of repetitive elements in leukocytes and buccal cells, as well as altered methylation at the CpG level in specific genes [57-60]. The biological effects of DNA methylation induced by exposure to air pollution have been investigated in the context of lung cancer risk [61], but little is known about their impact on breast cancer risk.

### Objectives

The overall objective of the XENAIR project is to investigate the chronic long-term effects of exposure to multiple ambient air pollutants and risk of breast cancer in a nested case-control study within the ongoing French prospective E3N (Etude Epidémiologique auprès des femmes de la Mutuelle Générale de l’Education Nationale) cohort. More specifically, the project aims to assess the associations between chronic exposure to selected ambient air pollutants (particulate matter, NO_2_, O_3_, BaP, dioxins, PCB 153, and cadmium) estimated from individual residential addresses of study subjects from recruitment (1990) and breast cancer risk. The study will (1) analyze exposure trajectory profiles of individual compounds over time and estimate breast cancer risk associated with each of these exposure profiles; (2) estimate breast cancer risk associated with the weighted cumulative duration of urban residence since birth (used as a surrogate for exposure to ambient air pollutions), as well as indoor exposure from domestic heating and combustion activities; (3) explore approaches to estimate multi-pollutant exposure.

The XENAIR project will further assess potential interactions between long-term exposure to low doses of air pollutants and genetic polymorphisms involved in air pollutant metabolism, to address the hypothesis that breast cancer risk associated with pollutants may depend on individual genetic susceptibility. We will also explore the potential role of DNA methylation as a marker of exposure to ambient air pollutions and as a potential mediator of the effect of ambient air pollutions on breast cancer risk. Additionally, we will estimate the fraction of breast cancers attributable to air pollution in France based on the risk estimates and additional costs of breast cancer management attributable to air pollutants.

## Methods

### The French E3N Cohort Study

E3N is an ongoing prospective cohort study launched in 1990 to investigate the main risk factors for cancer and severe chronic conditions in women [62]. Participants were recruited between June 1990 and November 1991 among women aged 40-65 years, living in France and insured with the MGEN, a national health insurance plan covering people working with the French education system and their families, and have been biennially followed-up with self-administered mailed questionnaires. E3N is the French part of the European Prospective Investigation on Cancer (EPIC), a vast European study coordinated by IARC and involving nearly 500,000 Europeans in 10 countries [63]. At recruitment, 98,995 E3N participants filled in a self-administered questionnaire, which included data about lifestyle and reproductive factors, anthropometry, past medical history, and familial history of cancer. To date, twelve questionnaires have been sent to the participants (participation rate at each questionnaire ~80%). Between 1994 and 1998, participants were invited to give a blood specimen. Blood samples were collected from 25,000 women, and saliva samples were later collected from an additional 47,000 women. The occurrence of cancer was self-reported in each questionnaire, and a small number of cancers were further identified from the insurance files or information on causes of death obtained from the National Service on Causes of Deaths. Pathology reports confirming diagnoses of invasive breast cancer (the primary outcome of the present project) were obtained for 93% of self-declared cases, and the proportion of false-positive self-reports was low (<5%). Addresses were recorded at baseline (1990) and the 5- and 9-year follow-up questionnaires (years 1997, 2000, 2002, 2005, 2008, and 2011). Postal codes were recorded at 3- and 4-years follow-up (1993 and 1994). Participants’ place of birth (postal code and municipality) was obtained from the first questionnaire and assigned an urban/rural status based on data from the closest national census [64]. Informed consent was obtained from each participant, and the study was approved by the French National Commission for Data Protection and Privacy (CNIL).

### Covariate Assessment

Data on established and potential breast cancer risk factors were available from the self-administered questionnaires at baseline. Regular updates have been collected on smoking, anthropometry (height, weight), physical activity, diabetes, hypertension, benign breast disease, gynecological screening, family history of breast cancer (FHbreast cancer), education, and reproductive factors. Women completed two validated self-administered diet history questionnaires (DHQ) in 1993 and 2005. The E3N DHQ covered the daily consumption of 208 food items by collecting food frequencies and portion sizes for eight meals and snacks during the day [65,66]. Dietary exposure to BaP, dioxins, PCB 153, and cadmium will be assessed for each woman by combining consumption data from the E3N DHQ and food contamination data available for France. Contamination data for BaP, dioxins, PCB 153, cadmium are available from the French agency for food safety (ANSES) [67,68].

### Study Population

The present study is based on a nested case-control subset of the E3N cohort. It involves 5222 histologically confirmed incident breast cancer cases identified during the 1990-2011 follow-up period. Women were included if they had completed their home address at baseline, lived in the French metropolitan territory during the 1990-2011 follow-up time, and not had any cancer at baseline. For each breast cancer case, one control was randomly selected by incidence density sampling, among cohort participants at risk of breast cancer at the time when the case was diagnosed, using the follow-up time since inclusion into the cohort as the time axis. In order to best select appropriate controls according to the planned studies, two complementary groups of cases were set, according to the presence of a blood sample, saliva sample, or no biological sample available. For the first group of cases (with a blood sample), controls were matched to cases on the department of residence, age (±1 year), date (±3 months), and menopausal status at blood collection. Controls for the second group (without a blood sample) were matched on the same criteria but collected at baseline, and additionally matched on the existence or not of a saliva sample.

### Additional Data Collection: Residential History Questionnaire Collection and Assessment of Indoor Air Pollution

A structured questionnaire was sent to all selected cases and controls to collect lifetime residential addresses from date of birth to the present (street address, municipality/city, postal code), school and workplace addresses, commute duration ( “less than 30 min,” “between 30 and 60 min,” “more than 60 min”) and type (“walking,” “cycling,” “motorcycle riding,” “driving a diesel car,” “driving a gasoline car,” or “using public transportation”), and information on domestic heating and combustion activities. We also collected information on the age when starting and stopping living in each reported home. Women were asked to report the period during which their home was built (before 1948, between 1948 and 1974, or after 1974), and whether it overlooked a courtyard or a street (courtyard, street, or both). In terms of indoor heating, they reported their main type of heating (collective central, or individual) and their main source of heating (wood, charcoal, electricity, gas, or fuel). Regarding indoor wood-burning stove or fireplace cooking, women were asked whether they used an indoor wood-burning stove or a fireplace in their home (yes, no), and if yes, the type (stove/wood stove, open fireplace, or closed fireplace). Information on the use and frequency of cooking foods on the barbecue was also collected (never, rarely (once to twice per year)), occasionally, or frequently (at least once a month)). Study participants additionally answered questions on whether they burned green waste (yes, no), and if yes the frequency (never, rarely (once or twice a year, occasionally, or frequently (at least once a month)), and the quantity of green waste burned every year (less than 1 m^3^, 1-5 m^3^, or more than 5 m^3^). The overall response rate was 65.4%.

### Geocoding of Residential History and Industrial Sources

The methods of geocoding residential history and industrial sources have been described in detail elsewhere [69]. Briefly, residential histories from the E3N follow-up questionnaires and the residential questionnaire will be geocoded (X and Y coordinates, addresses) using ArcGIS Software (ArcGIS Locator version 10.0, Environmental System Research Institute) and the national addresses database from the National Geographic Institute (BD Adresse, IGN). Geocoding will be performed by a trained technician blinded to the case-control status of the participants.

### Air Quality Modeling

Assessment at the national level of exposure to selected pollutants (PM_10_, PM_2.5_, NO_2_, O_3_, BaP, dioxins, PCB 153, and cadmium) will be based on complementary models according to data availability and pollutant emissions characteristics (Table 1). Specifically, we will use a regional chemistry-transport model (CHIMERE) [70], an urban gaussian dispersion model (SIRANE) [71], a land-use regression model [72] and a GIS-based metric [73]. A detailed description of these models is provided in Multimedia Appendix 1.

**Table 1:** Summary of the models used to assess atmospheric exposure.

Type of model (name)	Spatial and temporal resolution	Years	Area	Output/Goal	Pollutants
Eulerian chemistry-transport model (CHIMERE)	7 × 7 km, hourly	1990-2011	National (France)	Concentration/Exposure assessment	NO_2_, PM_2.5/10_, O_3_, cadmium, dioxins, PCB 153, BaP
National LUR^a^ model	50 × 50 m, annually	2010-2012	National (France)	Concentration/Exposure assessment	NO_2_, PM_2.5/10_, O_3_
Back-extrapolated national LUR model	50 × 50 m, annually	1990-2009	National (France)	Concentration/Exposure assessment	NO_2_, PM_2.5/10_, O_3_
GIS^b^-based metric	At the subject address, hourly	1990-2011	National (France)	Exposure Metric/Exposure assessment	cadmium, dioxins
Urban dispersion model (SIRANE)	10 × 10 m, hourly	1990, 1995, 2000, 2005, 2010	Local (Lyon)	Concentration/Sensitivity analysis	NO_2_, PM_2.5/10_, O_3_
Local LUR model	50 × 50 m, annually	2010	Local (Lyon)	Concentration/Sensitivity analysis	NO_2_
Back-extrapolated local LUR model	At the subject address, annually	1990, 1995, 2000, 2005,	Local (Lyon)	Concentration/Sensitivity analysis	NO_2_

^a^LUR: land-use regression.

^b^GIS: geographic information system.

#### PM10, PM2.5, NO2, and O3

We will use land-use regression models to estimate PM_10_, PM_2.5_, NO_2_, and O_3_ concentrations at the local scale (50 × 50 m) and develop “hybrid” models combining outputs from CHIMERE (concentrations over the whole French territory from 1990 to 2011, with a spatial resolution of 0.125° × 0.0625°) and localized variables describing road traffic and land use, nationwide. A so-called “baseline land-use regression model” will be constructed based on average measurement of 2010-2012 to ensure that meteorological conditions in a particular year do not bias predictions for other years. In this manner, we will also benefit from the largest quantity and the best quality of measurement data. This model will be validated against measurement across France by performing a hold-out validation (ie, independent monitoring sites). Once established, this model will be back-extrapolated until 1990. However, this step will benefit from the CHIMERE modeling results that will provide local concentrations from 2011 to 1990 to help adjust the back-extrapolation.

#### Dioxins and Cadmium

Ambient air concentration measurements of dioxins and cadmium are extremely fragmented and, therefore, it is difficult to estimate exposures. In addition, these measurements are unevenly distributed over time and space with an increasing number of measurements from the 2000s onwards, while at the same time, emissions are falling sharply, precluding the use of land-use regression models. Since dioxins and cadmium emissions over the period were mainly due to industrial sources and given the size of the study area, the use of dispersion models over the entire territory would not provide a sufficient spatial and temporal resolution to characterize exposure. To estimate dioxins and cadmium exposure at any point in the national territory over the 1990-2011 period, we adopt instead the approach used in a previous epidemiological study, ie, a GIS-based metric [74]. The latter was validated by comparison with a dispersion model in multiple contexts and is based on a detailed emission inventory [73].

#### BaP and PCB 153

Unlike dioxin and cadmium, no detailed emission inventory is currently available at a local scale for BaP and PCB 153. As a result, background concentrations from CHIMERE will be directly used as the reference concentrations for these compounds. BaP concentrations were already simulated with CHIMERE by Guerreiro et al [75], whereas PCB-153, cadmium, and dioxins are added into the model.

#### Sensitivity Analysis of Concentration Modeling

A sensitivity analysis will be done to compare the ability of different models to classify subjects correctly according to their exposures. The performance of the models will be compared to each other and with measurement data in ambient air. One of the most important objectives will be to quantify misclassification created by using a national model to assess exposures (see Multimedia Appendix 1).

#### Statistical Methods and Power Calculation

Associations with breast cancer risk will be modeled using conditional logistic regression models, considering different concentrations for each compound, and fuel sources for indoor heating and cooking. Exposure variables will be investigated as continuous and categorical variables, and models will be conditioned on the matching factors. All analyses will be adjusted for potential confounding and known breast cancer risk factors available from the self-administered questionnaires. Simple imputation methods will be used for missing continuous data, and a category of missing data will be created for categorical covariates.

Potential effect modification by follow-up time, age, BMI, tobacco smoking status, alcohol consumption, reproductive factors, and birthplace status will be tested using tests for interaction (likelihood ratio test). Further subgroup analyses will be conducted according to tumor hormone receptor status (ER and PR) and menopausal status. Heterogeneity of associations across hormone receptor subgroups will be assessed using polytomous logistic regression models [76].

The potential non-linearity of the relationship between exposures and breast cancer risk will be examined using restricted cubic splines [77] or fractional polynomials. In order to reduce residual confounding, potential non-linearity of the effects of continuous confounders will be accounted for using the same approach [78,79].

B-spline functions will be used in logistic regression models to estimate (1) the relative weight of the exposure dose versus time since recruitment or age at exposure and (2) breast cancer risk associated with the weighted cumulative duration of urban residence since birth (a surrogate for urban air pollutant exposure) [80,81].

To identify exposure trajectory profiles of individual compounds over time since recruitment in the cohort E3N, and to estimate breast cancer risk associated with each of these exposure profiles, we will use joint latent class mixed models [82].

Finally, different approaches will be explored in order to assess multipollutant exposure (Bayesian modeling, principal component analysis) [51,83].

For sensitivity analyses, models will be adjusted for estimated dietary exposure to each pollutant, considering the diet as a route of exposure separate from inhalation.

Table 2 presents different scenarios considered to calculate the statistical power to detect an association between a binary exposure (high versus low level) and the risk of breast cancer, using the power analysis method for matched case-control studies and a 5% type I error [84]. Overall, even for a low exposure prevalence of 20% and a case-control correlation of 0.2, we will have 97% power to detect an odds ratio of 1.2, and 100% power to detect an odds ratio of 1.5.

**Table 2:** Statistical power and sample size calculation.

Probability of exposure among controls	Correlation of exposure between matched controls and cases	Odds ratio adjusted for the matching variables	Power
0.2	0.5	1.2	0.85
0.2	0.5	1.5	1.00
0.2	0.2	1.2	0.97
0.2	0.2	1.5	1.00
0.5	0.5	1.2	0.96
0.5	0.5	1.5	1.00
0.5	0.2	1.2	0.99
0.5	0.2	1.5	1.00

#### Gene × Environment Interaction and DNA Methylation Analyses

To explore gene × environment interactions, we will first use a case-only study design, similar to Saintot et al [55], to analyze the interaction of exposure with single nucleotide polymorphisms in the metabolic pathways of dioxins, PCBs, and PAHs (cytochrome P450, glutathione S-transferase, and related pathways), growth factor, and inflammation pathway genes, in 2500 cases from a previous breast cancer study. A second analysis will be done in a nested case-control subset of 2500 cases and 2500 matched controls. DNA methylation analyses will be based on at least 400 case-control pairs with controls matched for age at recruitment, age at diagnosis of the corresponding case, and type of biospecimen (blood or saliva). These methods have been described in Multimedia Appendix 1.

#### Attributable Fraction and Cost Analyses

The Levin formula will be used to estimate the attributable fraction in the French general population, using our ORs estimates and nationwide exposure estimates [85]. Adopting the French national insurance perspective, direct costs (ie, those associated with diagnosis, surgery, chemotherapy, radiotherapy, and/or hormone therapy, and follow-up +/- relapse) of breast cancer attributable to ambient air pollution exposure will be assessed based on systematic reviews, observational and modeling studies, and expert opinion [86,87]. Costs will be combined with estimated attributable fractions to assess breast cancer treatment costs attributable to air pollutants in France.

### Ethics Approval and Consent to Participate

Our research is based on the E3N French national cohort. Informed consent was obtained from all participants, and the study was approved by the French National Commission for Data Protection and Privacy (CNIL).

## Results

The study is still ongoing. XENAIR will provide relevant and innovative evidence to fill gaps regarding the complex association of breast cancer risk with long-term exposure to multiple air pollutants (PM_2.5_, PM_10_, NO_2_, O_3_, dioxins, PCB 153, cadmium, and BaP) from 1990 to 2011, using complementary models (land-use regression and atmospheric dispersion models) at a fine spatio-temporal resolution. Our research will contribute to improving our understanding of life-long exposure and exposure at different life stages to urban settings. In addition, the investigation of gene × environment interactions will allow the identification of groups of women with genetic susceptibility to environmental carcinogens, and thus improve our understanding of the interaction of individual susceptibilities with environmental exposure. Furthermore, the identification of methylation markers of exposure to environmental pollutants will contribute to extend our understanding of breast cancer etiology and reveal biomarkers of exposure.

## Discussion

This study is prompted by the increasing incidence of breast cancer, although leveling off in recent years, persistent air pollution levels worldwide, and suggestive evidence for an association of breast cancer risk with several ambient air pollutants. To our knowledge, the XENAIR project is one of the largest prospective studies to investigate exposure to ambient air pollution and breast cancer risk, and it should significantly increase current knowledge on the health effects of air pollution. Investigating the impact of environmental exposure on breast cancer risk requires large studies with well-defined exposure information, as well as individuals’ risk factors and potential confounders.

Our research is based on the existing French national cohort, E3N [62]. This prospective cohort study is particularly well documented, with updated information every 2 years on established breast cancer risk factors and past medical history. The availability of detailed information on lifestyle factors, family history of breast cancer, and reproductive factors will allow for better control of confounding factors and further investigation of potential effect modifiers. Also, further Molecular pathological epidemiology analyses will be conducted according to hormone receptor status (ER and PR) of breast cancer. Detailed classification of tumor subtypes and their analyses will allow phenotype refinement, improve the identification of specific air pollution risk factors, and characterize the pathogenic molecular mechanisms of breast cancer. The Molecular pathological epidemiology research paradigm provides novel insights into interactions among environment, tumor, and host but also provides an exemplary model of integrative scientific approaches and contributes to advancements in precision medicine, therapy, and prevention [40].

Furthermore, because women from the E3N cohort are mostly teachers or have affiliated occupations, with potentially negligible occupational exposure, bias related to occupational exposures to the selected pollutants will be avoided. Since exposure to dioxins, cadmium, PCB 153, and BaP in the general population occurs through the ingestion of contaminated food and inhalation, the availability of consumption data from the E3N dietary questionnaires available for these compounds will allow further adjustment for dietary exposure [88]. Inhalation is the only route of exposure to PM_10_, PM_2.5_, NO_2_, and O_3_ in humans that is relevant to health effects; accordingly, we do not expect confounding from dietary exposure to play a role in exposure to these pollutants. The XENAIR project will also benefit from the large dioxin and cadmium sources inventories (1990-2011) [43,89] as well as the previously developed GIS-based metric [73]. By evaluating the transferability of the ESCAPE land-use regression models [90] to predict air pollution concentrations in large areas in France, XENAIR will contribute to the development of these technological advances for the assessment of long term exposure to air pollutants. One of the major strengths of our study is, however, the combination of CHIMERE and measurements to do back-extrapolation rather than measurements alone.

The use of GIS-based methods, combined with national-scale land-use regression and air dispersion models at different spatial and temporal resolutions, will help to better describe environmental pollutant exposure better. The large dataset created by thorough geocoding of subjects’ residential history [69] will enable integrative analyses of additional environmental risk factors. Findings from our research will create a basis for refined assessments of the impact of exposure to air pollution on other diseases within the E3N cohort or other national cohort studies.

Limitations of the XENAIR project include the lack of available historical exposure data before 1990, making it impossible to have a complete individual lifetime dose estimate for the E3N women; however, investigating lifetime urban/rural status will partially remediate this limitation. The spatial resolution of the PCB153 and BaP model will be limited to 7 km^2^, which may not be sufficient to describe airborne exposure in dense urban contexts. From a more global perspective, the assessment of historical airborne pollutant exposure will create more uncertainties that must be taken into account. We may minimize the impact of these biases by using different approaches to exposure assessment.

In conclusion, XENAIR will create a large-scale, national dataset on multiple ambient air pollutant exposures and contribute to a better understanding of environmental modifiable risk factors related to breast cancer. The results of our interdisciplinary research will contribute to the concept of the exposome [50] at the individual and societal levels, and provide support to governments and policy-makers to better design effective public health prevention strategies and to promote urban policies in order to reduce exposure to ambient air pollution further. The XENAIR dataset will enable future investigations of the effects of exposure to air pollution associated with other diseases.

## Appendix

Multimedia Appendix 1 Detailed description of the different models used for the pollutants exposure assessment.

## Abbreviations

ANSES: French agency of food safety
BaP: Benzo[a]pyrene
CNIL: commission for data protection and privacy
DHQ: diet history questionnaires
ER: estrogen receptor
E3N: Etude Epidémiologique auprès des femmes de la Mutuelle Générale de l’Education Nationale
EPIC: European Prospective Investigation into Cancer and Nutrition
GIS: geographic information system
GWAS: genome-wide association study
IARC: International Agency for Research on Cancer
IGN: National Geographic Institute
LMA: Lyon metropolitan area
LUR: land-use regression
MHT: menopausal hormone therapy
PAH: polycyclic aromatic hydrocarbon
PCBs: polychlorinated biphenyls
PR: progesterone receptor
