# Supplementary material for: Chronic Low-Dose Exposure to Xenoestrogen Ambient Air Pollutants and Breast Cancer Risk: XENAIR Protocol for a Case-Control Study Nested Within the French E3N Cohort
Source: JMIR Res Protoc. 2020 Sep 15;9(9):e15167. doi: 10.2196/15167 (PMC7525465; doi:10.2196/15167)
Supplement: Multimedia Appendix 1 [file resprot_v9i9e15167_app1.doc]

**Chronic low-dose exposure to xenoestrogen ambient air pollutants and risk of breast cancer: study protocol of XENAIR Project**

Amina Amadou ^1 9^, Thomas Coudon ^1 3^, Delphine Praud ^1 2 9^ , Pietro Salizzoni ^3^ , Karen Leffondré ^5^, Emilie Lévêque ^5^, Marie-Christine Boutron-Ruault ^6^, Aurélie MN Danjou ^4^, Xavier Morelli ^1^, Charlotte Le Cornet ^1 10^, Lionel Perrier ^1^, Florian Couvidat ^7^, Bertrand Bessagnet ^7^, Julien Caudeville ^7^, Elodie Faure ^1^, Francesca Romana Mancini ^6^ John Gulliver ^8^, Gianluca Severi ^6^ and Béatrice Fervers ^1 9^

^1^ Department of Prevention Cancer Environment, Centre Léon Bérard, Lyon, France

^2^ Inserm U1052, CNRS UMR 5286, Univ. Lyon 1, Cancer Research Center of Lyon, Lyon, France

^3^ Ecole Centrale de Lyon, INSA Lyon, Université Claude Bernard Lyon 1, Ecully, France

^4^ Section of Environment and Radiation, International Agency for Research on Cancer (IARC)

^5^ Université de Bordeaux, ISPED, Inserm U1219, Bordeaux Population Health Center, Bordeaux, France

^6^ Centre de Recherche en Epidémiologie et Santé des Populations (CESP, Inserm U1018), Faculté de Médecine, Université Paris-Saclay, UPS UVSQ, Gustave Roussy, Villejuif, France

^7^ National Institute for industrial Environment and Risks (INERIS), Verneuil-en-Halatte 60550, France;

^8^ Centre for Environmental Health and Sustainability, School of Geography, Geology and the Environment, University of Leicester, United Kingdom.

^9^ Inserm UA 08 Radiations : Défense, Santé, Environnement, F-69008 Lyon, France

^10^ Division of Cancer Epidemiology, German Cancer Research Center (DKFZ), Heidelberg, Germany

**Corresponding author**

Béatrice Fervers

[beatrice.fervers@lyon.unicancer.fr](mailto:beatrice.fervers@lyon.unicancer.fr)

Prevention Cancer Environnement Department, Centre Léon Bérard

Inserm UA 08 Radiations : Défense, Santé, Environnement,

28 rue Laënnec, 69373 Lyon Cedex 08, France.

Phone: +33(0)478782801- Fax:+33.(0)

Gianluca Severi

[severi.gianluca@gustaveroussy.fr](mailto:severi.gianluca@gustaveroussy.fr)

Centre de Recherche en Epidémiologie et Santé des Populations (CESP, Inserm U1018)

Faculté de Médecine, Université Paris-Saclay, UPS UVSQ, Gustave Roussy

114 rue Edouard-Vaillant, 94805 Villejuif Cedex, France

Phone: +33(0)142115864

**ABSTRACT**

**BACKGROUND:** Breast cancer (BC) is the most frequent cancer in women in industrialized countries. Lifestyle and environmental factors, particularly endocrine disrupting pollutants have been suggested to play a role in BC risk. Current epidemiological studies, although not fully consistent, suggest a positive association of BC risk with exposure to several International Agency for Research on Cancer Group 1 air pollutant carcinogens, such as particulate matter (PM), polychlorinated biphenyls (PCB), dioxins, Benzo[a]pyrene (BaP), and cadmium. However, overall epidemiological studies are still scarce and inconsistent. It has been proposed that the menopausal status could modify the relationship between pollutants and BC, and that the association could differ according to the BC hormone receptor status.

**OBJECTIVE**: The XENAIR project will investigate the association of BC risk (overall and by hormone receptor status) with chronic exposure to selected air pollutants (PM, NO_2_, O_3_, BaP, dioxins, PCB153, and cadmium).

**METHODS**: Our research is based on a case-control study nested within the French national E3N cohort involving 5,224 incident invasive BC cases identified during follow-up from 1990 to 2010, and 5,224 matched controls. A specific questionnaire was sent to all participants to collect their lifetime residential addresses and information on indoor pollution. We will assess these exposures using complementary models (land-use regression, atmospheric dispersion, CHIMERE models) via a Geographic Information System. Associations with BC risk will be modelled using conditional logistic regression models. We will further study the impact on DNA methylation and interactions with genetic polymorphisms. Appropriate statistical methods, including Bayesian modelling, principal component analysis, and cluster analysis, will be used to assess the impact of multipollutant exposure. The fraction of BC cases attributable to air pollution will be further estimated.

**RESULTS**: The XENAIR project will contribute to increase current knowledge on the health effects of air pollution, and to better identify and understand environmental modifiable risk factors related to BC risk.

**CONCLUSIONS**: The results will provide relevant evidence to governments and policy-makers to improve effective public health prevention strategies on air pollution. The XENAIR dataset can be used in future studies to investigate the effects of exposure to air pollution associated with other chronic conditions.

**Keywords:** breast cancer; hormone receptor status; air pollution; endocrine disruptors; multi-pollutant; geographic information system; land use regression; chemistry-transport model; epigenetic; gene-environment interaction; prospective study

**INTRODUCTION**

**Background**

Breast cancer (BC) is the most common cancer among women worldwide, with an estimated 2.09 million new cancer cases diagnosed in 2018 in the world [1]. Over the past thirty years, its incidence has continuously increased in France [1,2]. The rapid increase in the incidence rates worldwide has been associated with mass screening, menopausal hormonal therapy, and societal changes impacting individuals’ lifestyles. Epidemiological studies have suggested an important role of lifestyle and environmental factors, supported by geographical variations of BC incidence and time trends in incidence rates among migrant populations [3–5]. However, a considerable proportion of BC risk remains to be explained and the impact of environmental factors in the etiology of BC has not been fully explored to date. Epidemiology and laboratory findings suggest that exposures to environmental pollutants, in particular those with potential estrogenic effects, may play a role in BC development [6,7].

Ambient air pollution (AAP) is a major public health concern related to a range of adverse health effects, including cancer, and accounting for an estimated 4.2 million deaths per year [8]. In 2013, the International Agency for Research on Cancer (IARC) classified outdoor air pollution as a whole, as well as particulate matter (PM), as carcinogenic to humans, principally based on studies on lung and bladder cancers [9]. However studies on BC are scarce and results remain inconsistent [10]. A recent meta-analysis of individual data from 15 European cohorts revealed no association between postmenopausal BC and exposure to PM_2.5_, PM_10_, PM coarse; however they reported a statistically significant positive relationship per 20 µg/m^3^ increase in nitrogen dioxide (NO_2_) exposure [11]. Epidemiological evidence suggests an association between BC and NO_2_ from traffic-related air pollution [12–15]. Furthermore, it has been reported that women with extremely dense mammography density, a well-established risk factor for BC, were less likely to have higher levels of ozone (O_3_) exposure [16]. BC has also been positively linked to exposures to pollutants with endocrine disrupting properties, such as polychlorinated biphenyls (PCBs) [17,18], dioxins [19–21], benzo[a]pyrene (BaP) [12,22], and cadmium [23,24] with sometimes diverging results. . Furthermore, studies have reported positive associations between traffic-related BaP exposure, as a surrogate for Polycyclic aromatic hydrocarbon (PAH) exposure and BC [25,26]. Cadmium has been classified by IARC as carcinogenic to humans (Group 1), with sufficient evidence for lung cancer [27]. However, the only study performed on airborne cadmium exposure and BC risk found no evidence for overall increased BC risk, however, elevated risks for hormone receptor-negative tumors (estrogen receptor and progesterone receptor negative (ER-PR-)) were observed for higher exposure to cadmium [28].

Indoor pollution is also an important challenge for global health. Around 3 billion people use traditional biomass fuels for household cooking and heating [29] , which is a major source of indoor air pollution, and source of dioxins, PAH, PM, O_3_, and NO_2_ exposure [30–35]. However, only two studies have investigated the impact of indoor pollution on BC risk [36,37]. Overall, current epidemiological evidence regarding the impact of exposure to AAP and indoor pollution on BC risk is not fully consistent. In addition, joint exposures to multiple compounds have been insufficiently explored. Also, evidence supporting differential effects on BC risk by menopausal status and according to the BC hormone receptor status remains limited [23]. Therefore, further research is needed to investigate whether the associations vary by women menopausal status and BC hormone receptor status. Currently, BC is no longer considered as a homogeneous disease, but a heterogeneous disease composed of several distinct molecular subtypes according to their hormone receptor status (ER, PR) and human epidermal growth factor receptor 2 (HER2) [38]. These subtypes each have different prognoses and can affect women differently. Molecular pathological epidemiology (MPE), integrating molecular pathology into epidemiological studies has emerged in order to test for a difference between the association of a specific environmental exposure with subtypes classified by molecular features in determining disease incidence/mortality [39,40].

One of the major limitations of previous studies is the lack of past residential history and/or historical AAP exposure estimates, which may have resulted in exposure misclassification, likely contributing to imprecise risk estimates and bias towards the null [41]. Many epidemiological studies have relied on a variety of exposure assessment techniques such as using data from centrally located ambient air quality monitoring networks, surrogates for exposures. However, such methods are often insufficient for capturing the spatial variability of pollutant concentrations at the local scale, both at the intra-urban scale, and the suburban and rural scale [42]. More complex techniques have been used in recent studies to adequately represent the spatial-temporal variation of pollutants, including land use regression (LUR) models, dispersion modelling (DM), chemistry transport models (CTMs), and hybrid models [43]. The combination of these complexes methods will allow to assess the exposure to each pollutant of the study at a fine spatial scale and over a large areas between 1990 and 2010.

**Life course trajectories**

Exposures occurring early in life, and/or during biological windows of greater sensitivity (i.e. in utero and during childhood) have been suggested to be more strongly associated with BC risk [44,45]. Yet, very few studies have investigated these effects, and the majority of current research was based on adulthood exposures within short observation periods. These studies may have missed critical windows and/or cumulative effects of lifetime exposure that could impact BC risk [45,46]. Lack of historical measures makes retrospective exposure reconstruction difficult, especially for earlier periods. A higher BC incidence is commonly observed in large cities compared with rural areas [47]. Also, being born in an urban area was related to an increased BC risk compared with women born in rural areas [47]. To our knowledge, no study, to date, has investigated the effects of life-course residential trajectories on BC. Urban residence has been suggested to be a surrogate of air pollution exposure released from road traffic, industrial facilities, and waste incineration [48], and to be useful to investigate earlier periods where historical air pollution records are unavailable and back-extrapolation unfeasible [47].

**Cumulative and multiple exposures**

Early evidence suggests that cancer risk may not be a linear function of cumulative carcinogen dose [49]. Individuals are exposed simultaneously to a complex and changing mixture of environmental exposures [50]. These may independently, cumulatively or interactively influence the risk of developing BC. Furthermore, in single pollutant models, it is unclear whether an observed association is due to the effect of the evaluated pollutant or whether it acts as a surrogate for another pollutant from the same source. Multipollutant approaches need to address the complex structure of mixtures that frequently present multicollinearity. However such epidemiological studies are limited [51,52].

**Gene-environment interactions**

Current evidence on the role of genetic susceptibility (polymorphisms) related to exposure to AAP remains limited. Previous epidemiological studies have reported interactions between genetic polymorphisms and some pollutants [53,54]. Saintot et al, , reported that women carrying the Val CYP1B1 allele and who had lived near to a waste incinerator for more than 10 years had a higher risk of BC than those never exposed with the Leu/Leu genotype [55]., A positive association was found in postmenopausal women with a CYP1A1 variant genotype [56].

**DNA methylation**

Methylation is one of several epigenetic events involved in the regulation of gene expression, and it can undergo alterations as a consequence of environmental stimuli. Emerging evidence suggests that exposure to AAPs could influence DNA methylation, producing hypomethylation of repetitive elements in leukocytes and buccal cells, as well as altered methylation at the CpG

level in specific genes [57–60]. The biological effects of DNA methylation induced by exposure to air pollution have been previously investigated in relation to lung cancer risk [61], but little is known about their impact on BC risk.

**Objectives**

The overall objective of the XENAIR project is to investigate chronic long-term effects of exposure to multiple AAPs and risk of BC in a nested case-control study within the ongoing French prospective E3N (Etude Epidémiologique auprès de femmes de la Mutuelle Générale de l’Education Nationale) cohort. More specifically, the project aims at assessing associations between chronic exposure to selected AAPs (PM, NO_2_, O_3_, BaP, dioxins, PCB 153, and cadmium) estimated from individual residential addresses of study subjects from recruitment (1990) and BC risk. The study will particularly: i) analyze exposure trajectory profiles of individual compounds over time since recruitment, and estimate BC risk associated with each of these exposure profile; ii) estimate BC risk associated with the weighted cumulative duration of urban residence since birth (used as a surrogate for exposure to AAPs), as well as with indoor exposure from domestic heating and combustion activities; iii) explore approaches to estimate multi-pollutant exposure.

The XENAIR project will further assess potential interactions between long-term exposure to low doses of air pollutants and genetic polymorphisms involved in air pollutant metabolism, to address the hypothesis that BC risk associated with pollutants may depend on individual genetic susceptibility. We will also explore the potential role of DNA methylation as a marker of exposure to AAPs and as a potential mediator of the effect of AAPs on BC risk. Additionally, we will estimate the fraction of BCs attributable to air pollution in France based on the risk estimates, and the additional costs of BC management attributable to air pollutants.

**METHODS**

**The French E3N cohort study**

E3N is an ongoing prospective cohort study launched in 1990 to investigate the main risk factors for cancer and severe chronic conditions in women [62]. Participants were recruited between June 1990 and November 1991 among women aged 40-65 years, living in France and insured with the MGEN, a national health insurance plan covering people working with the French education system and their families, and have been biennially followed-up with self-administered mailed questionnaires. E3N is the French part of European Prospective Investigation on Cancer (EPIC), a vast European study coordinated by IARC and involving nearly 500,000 Europeans in 10 countries [63]. At recruitment, 98,995 E3N participants filled in a self-administered questionnaire, which included data about lifestyle and reproductive factors, anthropometry, past medical history, and familial history of cancer. To date, twelve questionnaires have been sent to the participants (participation rate at each questionnaire ~80%). Between 1994 and 1998, participants were invited to give a blood specimen. Blood samples were collected from 25,000 women, and saliva samples were later collected from an additional 47,000 women. Occurrence of cancer was self-reported in each questionnaire, and a small number of cancers were further identified from the insurance files or information on causes of death obtained from the National Service on Causes of Deaths. The pathology report, for confirming diagnoses of invasive BC (the primary outcome of the present project) was obtained for 93% of self-declared cases, and the proportion of false-positive self-reports was low (<5%). The addresses of the subjects selected for the study have been recorded at baseline (1990) and at the 5 to 9 follow-up questionnaires (years 1997, 2000, 2002, 2005, 2008, and 2011). Postal codes of participants were recorded at follow-up questionnaires 3 and 4 (1993 and 1994). In addition, participants’ place of birth (postal code and municipality) was obtained from the first questionnaire and assigned an urban/rural status based on data from the closest national census [64]. An informed consent was obtained from each participant, and the study was approved by the French National Commission for Data Protection and Privacy (CNIL).

**Covariate assessment**

Data on established and potential BC risk factors were available from the self-administered questionnaires at baseline. Regular updates have been collected on smoking, anthropometry (height, weight), physical activity, diabetes, hypertension, benign breast disease, gynecological screening, family history of breast cancer (FHBC), education, and reproductive factors. Women completed two validated self-administered diet history questionnaires (DHQ) in 1993 and 2005. The E3N DHQ covered the daily consumption of 208 food items by collecting food frequencies and portion sizes for 8 meals and snacks during the day [65,66]. Dietary exposure to BaP, dioxins, PCB 153, cadmium will be assessed for each woman, by combining consumption data from the E3N DHQ and food contamination data available for France. These contamination data are available from the French agency of food safety (ANSES) for BaP, dioxins, PCB 153, cadmium [67,68].

**Study population**

The present study is based on a nested case-control subset of the E3N cohort. It involves 5,224 histologically confirmed incident BC cases, identified during the 1990-2010 follow-up period. Women were included if they had completed their home address at baseline, lived in the metropolitan French territory during the 1990-2010 follow-up time, and not had any cancer at baseline. For each BC case, one control was randomly selected by incidence density sampling, among cohort participants at risk of BC at the time when the case was diagnosed, using the follow-up time since inclusion into the cohort as time axis. In order to best select appropriate controls according to the planned studies, two complementary groups of cases were set, according to presence of a blood sample, presence of a saliva sample, or no biological sample available. For the first group of cases (with a blood sample), controls were matched to cases on department of residence, age (± 1 year), date (± 3 months), and menopausal status at blood collection. Controls for the second group (without a blood sample) were matched on the same criteria but collected at baseline, and additionally matched on the existence or not of a saliva sample.

**Additional data collection: Residential history questionnaire collection and assessment of indoor air pollution**

A specific structured questionnaire was sent to all selected cases and controls to collect lifetime residential addresses from date of birth to present (street address, municipality/city, postal code), school and workplace addresses, duration ( “less than 30 min”, “between 30 and 60 min”, “more than 60 min”) and type of commutes (“walking”, “cycling”, “motorcycle riding”, “driving a diesel car”, “driving a gasoline car” or “using public transportation”), and information on domestic heating and combustion activities in each of the women’s residencies. We additionally collected information on age when starting and stopping living in each reported home. Women were asked to report the period their housing was built (before 1948, between 1948 and 1974, or after 1974), and whether it overlooked a courtyard or a street (courtyard, street, or both). In terms of indoor heating, they reported their main type of heating (collective central, or individual), their main source of heating (wood, charcoal, electricity, gas, or fuel). Regarding indoor wood-burning stove or fireplace cooking, women were asked whether they used an indoor wood-burning stove or a fireplace in their home (yes, no), and if yes, the type (stove/wood stove, open fireplace, or closed fireplace). Information on use and frequency of cooking foods on the barbecue was also collected (never, rarely (once to twice per year)), occasionally, or frequently (at least once a month)). Study participants additionally answered questions on whether they burned green waste (yes, no), and if yes the frequency (never, rarely (one or twice a year, occasionally, or frequently (at least once a month)), and the quantity of green waste burned every year (less than 1 m^3^, 1- 5 m^3^, or more than 5 m^3^). The overall response rate was 65.4%.

**Geocoding of residential history and industrial sources**

The methods of geocoding the residential history and industrial sources has been described in detail elsewhere [69]. Briefly, the residential history of the participants from the E3N follow-up questionnaires and the residential questionnaire will be geocoded (X and Y coordinates, addresses) at the address using the ArcGIS Software (ArcGIS Locator version 10.0, Environmental System Research Institute – ESRI, Redlands, CA, USA) and the national addresses database from the National Geographic Institute (BD Adresse®, IGN). Geocoding will be performed by a trained technician blinded to the case-control status of the participants.

**Air quality modelling**

Assessment at the national country level of exposure to selected pollutants (PM_10_, PM_2.5_, NO_2_, O_3_, BaP, dioxins, PCB 153 and cadmium) will be based on complementary models according to data availability and pollutants’ emissions characteristics (Table 1). Specifically, we will use a regional chemistry-transport model (CHIMERE) [70], an urban gaussian dispersion model (SIRANE) [71] a Land Use Regression model (LUR) [72] and a GIS based metric [73] A detailed description of these models is provided in additional file 1.

**Table 1** Summary of the models used to assess atmospheric exposure in the study

| Type of model (name) | Spatial & temporal resolution | Time cover | Area of application | | Output / Goal | Pollutants |
| --- | --- | --- | --- | --- | --- | --- |
| Eulerian chemistry-transport model (CHIMERE) | 7x7km,  Hourly | 1990-2010 | | National (France) | Concentration / Exposure assessment | NO_2_, PM_2.5/10_, O_3_, Cadmium, Dioxins  PCB 153, BaP |
| National LUR model | 50x50m, Annually | 2010-2012 | | National (France) | Concentration / Exposure assessment | NO_2_, PM_2.5/10_, O_3_ |
| Back-extrapolated national LUR model | 50x50m, Annually | 1990-2009 | | National (France) | Concentration / Exposure assessment | NO_2_, PM_2.5/10_, O_3_ |
| GIS based metric | At the subject address, Hourly | 1990-2010 | | National (France) | Exposure Metric / Exposure assessment | Cadmium, Dioxins |
| Urban dispersion model (SIRANE) | 10x10m Hourly | 1990, 1995, 2000, 2005, 2010 | | Local (Lyon) | Concentration /  Sensitivity analysis | NO_2_, PM_2.5/10_, O_3_ |
| Local LUR model | 50x50m, Annually | 2010 | | Local (Lyon) | Concentration /  Sensitivity analysis | NO_2_ |
| Back-extrapolated local LUR model | At the subject address, Annually | 1990, 1995, 2000, 2005, | | Local (Lyon) | Concentration /  Sensitivity analysis | NO_2_ |

*PM_10_, PM_2.5_, NO_2_ and O_3_*

We will use LUR models to estimate PM_10_, PM_2.5_, NO_2_ and O_3_ concentrations at the local scale (50x50m) and develop ‘hybrid’ models combining outputs from CHIMERE (concentrations over the whole French territory from 1990 to 2010, with a spatial resolution of 0.125° x 0.0625°) and localized variables describing road traffic and land use, nationwide. A so-called “baseline LUR model” will be constructed based on average measurement of 2010-2012 to ensure that predictions for other years are not biased by meteorological conditions in a particular year. In this manner, we will also benefit of the largest quantity and a best quality of measurement data. This model will be validated against measurement across France by performing a hold-out validation (i.e. independent monitoring sites). Once established, this model will be back-extrapolated until 1990. However, this step will benefit from the CHIMERE modeling results that will provide local concentrations from 2010 to 1990 to help adjusted the back-extrapolation.

*Dioxins and Cadmium*

Dioxins and cadmium ambient air concentration measurements are extremely fragmented and therefore difficult to estimate exposures. In addition, these measurements are unevenly distributed over time and space with an increasing number of measurements from the 2000s onwards, while at the same time emissions are falling sharply. This precludes the use of LUR models. Since dioxins and cadmium emissions over the period were mainly due to industrial sources, and given the size of the study area, the use of dispersion models over the entire territory would not provide a sufficient spatial and temporal resolution to characterize exposure. To estimate dioxins and cadmium exposure at any point in the national territory over the 1990-2010 period, we adopt instead the approach used in a previous epidemiological study, i.e. a GIS-based metric [74]. The latter was validated by comparison with a dispersion model in multiple contexts and is based on a detailed emission inventory [73].

*BaP and PCB 153*

Contrary to dioxin and cadmium, no detailed emission inventory is currently available at a local scale for BaP and PCB 153. As a result, background concentrations from CHIMERE will be directly used as the reference concentrations for these compounds. BaP concentrations were already simulated with CHIMERE by Guerreiro et al. [75] whereas PCB-153, cadmium and dioxins are added into the model.

**Sensitivity analysis of concentration modeling**

A sensitivity analysis will be done to compare the ability of different models to correctly classify subjects according to their exposures. The performance of the models will be compared to each other and with the measurements data in ambient air. One of the most important objectives will be to quantify the misclassification induce by the use of a national model to assess exposures (see appendix).

**Statistical methods and power calculation**

Associations with BC risk will be modelled using conditional logistic regression models, considering different concentrations for each compound, and indoor combustions. Exposure variables will be investigated as continuous variables as well as categorical variables. Models will be conditioned on the matching factors. All analyses will be adjusted for potential confounding and known BC risk factors available from the self-administered questionnaires. Simple imputation methods will be used for missing continuous data, and a category of missing data will be created for categorical covariates.

Potential effect modification by follow-up time, age, BMI, tobacco smoking status, alcohol consumption, reproductive factors, and birthplace status will be tested using tests for interaction (likelihood ratio test). Further subgroup analyses will be conducted according to hormone receptor status (ER and PR) of the breast tumors and women menopausal status. Heterogeneity of associations across hormone receptor subgroups will be assessed using polytomous logistic regression models [76].

The potential non-linearity of the relation between exposures and BC risk will be examined using restricted cubic splines [77] or fractional polynomials. In order to reduce residual confounding, potential non-linearity of the effects of continuous confounders will be accounted for using the same approach [78,79].

In addition, B-spline functions will be used in logistic regression models to estimate: i) the relative weight of the exposure dose with respect to time since recruitment or age at exposure; ii) BC risk associated with the weighted cumulative duration of urban residence since birth (surrogate for urban air pollutant exposure) [80,81].

To identify exposure trajectory profiles of individual compounds over time since recruitment in the cohort E3N, and to estimate BC risk associated with each of these exposure profiles, we will use joint latent class mixed models [82].

Finally, different approaches will be explored in order to assess multipollutant exposure (Bayesian modelling, principal component analysis) [51,83].

For sensitivity analyses, models will be additionally adjusted for estimated dietary exposure to each pollutant, considering the diet as a route of exposure besides inhalation.

Table 2 presents different scenarios considered to calculate the statistical power to detect an association between a binary exposure (high versus low level) and the risk of BC, using the power analysis method for matched case–control studies and a 5% type I error [84]. Overall, even for low exposure prevalence of 20% and a correlation of 0.2 between cases and controls, we will have a power of 0.97 to detect an OR of 1.2, and a power of 100% to detect an OR of 1.5.

***Table 2: Statistical power and sample size calculation***

| Probability of exposure among controls | Correlation of exposure between matched controls and cases | Odds ratio adjusted for the matching variables | Power |
| --- | --- | --- | --- |
| 0.2 | 0.5 | 1.2 | 0.85 |
|  |  | 1.5 | 1.00 |
|  | 0.2 | 1.2 | 0.97 |
|  |  | 1.5 | 1.00 |
| 0.5 | 0.5 | 1.2 | 0.96 |
|  |  | 1.5 | 1.00 |
|  | 0.2 | 1.2 | 0.99 |
|  |  | 1.5 | 1.00 |

**Gene-environment interaction & DNA methylation analyses**

To explore gene-environment interactions, we will first use a case-only study design, similar to Saintot et al. [55] to analyze interaction of exposure with Single Nucleotide Polymorphisms (SNPs) in metabolism pathways of dioxins, PCBs, and PAHs (CYP and GST genes and related pathways), growth factor, and inflammation pathway genes, among 2,500 cases already available from a previous BC study. A second analyze will be done in a nested case-control subset of 2,500 cases and 2,500 matched controls. The analyses of DNA methylation will be based on at least 400 case-control pairs with controls matched for age at recruitment, age at diagnosis of the corresponding case and type of biospecimen (blood or saliva). Detail of these methods have been described in the appendix.

**Attributable fraction and cost analyses.**

The Levin formula will be used to estimate the attributable fraction in the French general population, using our ORs estimates and nationwide exposure estimates [85]. Adopting the French national insurance perspective, direct costs (i.e. those associated with diagnosis, surgery, chemotherapy, radiotherapy, and/or hormone therapy, and follow-up +/- relapse) of BC attributable to AAP exposure will be assessed based on systematic reviews, observational and modelling studies, and expert opinion [86,87]. Costs will be combined with estimated AFs to assess BC treatment costs attributable to air pollutants in France.

**RESULTS**

The study is still on-going. XENAIR will particularly provide relevant and innovative evidence to fill the existing gaps regarding the complex association of BC risk with long-term exposure to multiple air pollutants (PM_2.5_, PM_10_, NO_2_, O_3_, dioxins, PCB 153, cadmium, and BaP) from 1990 to 2010, using complementary models (LUR and atmospheric dispersion models) at a fine spatio-temporal resolution. Our research will contribute to improve our understanding of life-long exposure and exposure at different life stages to urban settings. In addition, the investigation of gene-environment interactions will allow identifying groups of women with genetic susceptibility to environmental carcinogens, and thus improve our understanding of the interaction of individual susceptibilities with environmental exposure. Furthermore, the identification of methylation markers of exposure to environmental pollutants will contribute to extend our understanding of BC etiology and provide biomarkers reflecting exposures

**DISCUSSION**

The increasing incidence of BC although with a leveling off in the recent years, persistent air pollution levels worldwide, and suggestive evidence for an association of BC risk with several AAPs, stress the relevance of the present research. To our knowledge, the XENAIR project is one of the largest prospective studies to date investigating AAP exposure and BC risk, and it should significantly contribute to increase current knowledge on the health effects of air pollution. Investigating the impact of environmental exposure on BC risk requires large studies with well-defined exposure information, as well as individuals’ risk factors and potential confounders.

Our research is based on the existing French national cohort E3N [62]. This prospective cohort study is particularly well documented, with updated information every two years on established BC risk factors and past medical history. Availability of detailed information on lifestyle factors, FHBC and reproductive factors will allow for better control of confounding factors and further investigation of potential effect modifiers. Also, further MPE analyses will be conducted according to hormone receptor status (ER and PR) of BC. Detailed classification of tumour subtypes and their analyses will allow to refine phenotype, to improve the identification of specific air pollution risk factors, and to understand the molecular pathogenic mechanisms of BC. The MPE research paradigm is recognized to provide novel insights into interactions among environment, tumor, and host but also provides an exemplary model of integrative scientific approaches and contributes to advancements in precision medicine, therapy, and prevention [40]. Furthermore, because women from the E3N cohort are mostly teachers or have affiliated occupations, with potentially negligible occupational exposure, bias related to occupational exposures to the selected pollutants will be avoided. Since exposure to dioxins, cadmium, PCB 153, and BaP in the general population occurs through ingestion of contaminated food and inhalation, the availability of consumption data from the E3N dietary questionnaires available for these compounds will allow further adjustment for dietary exposure [88]. Concerning PM_10_, PM_2.5_, NO_2_, and O_3_, inhalation is the only route of exposure in humans that is relevant in relation to health effects, accordingly, we do not expect confounding from dietary exposure to play a role in exposure to these pollutants. The XENAIR project will also benefit from the large dioxin and cadmium sources inventories (1990-2010) [43,89] as well as the previously developed GIS-based metric [73]. By evaluating the transferability of the ESCAPE LUR models [77,95] to predict air pollution concentrations in large areas in France, XENAIR will contribute to further develop these technological advances for the assessment of long term exposure to air pollutants. One of the major strength of our study is, however, the combination of CHIMERE and measurements to do back-extrapolation, rather than measurements alone.

The use of GIS-based methods, in association with national-scale land-use regression and air dispersion models with different spatial and temporal resolutions, will help to better describe environmental pollutant exposure. The large dataset resulting from the thorough geocoding of residential history of study subjects [69] will allow integrating analyses of additional environmental risk factors. Findings from our research will therefore create a basis for refined assessments of the impact of exposure to air pollution on other diseases within the E3N cohort or other existing national cohort studies.

Limitations of the XENAIR project include the lack of available historical exposure data prior to 1990, making it impossible to have a complete individual lifetime dose estimate for the E3N women; however investigating lifetime urban/rural status aims to respond partly to this limitation. For PCB 153 and BaP, the spatial resolution of the model will be limited to 7x7km which may not be sufficient to correctly describe airborne exposure in dense urban contexts. In a more global perspective, assessment of historical airborne pollutant exposure will induce higher uncertainties that must be taken into account. We may minimize the impact of these bias by using different approaches of exposure assessment.

In conclusion XENAIR will create a large-scale, national dataset on multiple AAP exposures and contribute to better understanding environmental modifiable risk factors related to BC. The results of our interdisciplinary research will contribute to the concept of the exposome [50] at the individual and societal levels, and provide support to governments and policy-makers to better design effective public health prevention strategies and to promote urban policies in order to further reduce AAP exposure. The XENAIR dataset will enable future investigations of the effects of exposure to air pollution associated with other diseases.

**List of abbreviations**

AAP: ambient air pollution; AF: attributable fraction; ANSES: French agency of food safety; BC: breast cancer; BMI: body mass index; BaP: Benzo[a]pyrene; CTMs: chemistry transport models; CIs: confidence intervals; CNIL: commission for data protection and privacy; DM: dispersion modelling; DHQ: diet history questionnaires; ER: estrogen receptor; E3N: Etude Epidémiologique auprès de femmes de la Mutuelle Générale de l’Education Nationale; EPIC: European Prospective Investigation into Cancer and Nutrition; GIS: geographic information system; GWAS: genome-wide association study; IARC: international agency for research on cancer; IGN: National Geographic Institute; LMA: Lyon metropolitan area; LUR: land-use regression; MHT: menopausal hormone therapy; NO_2_: nitrogen dioxide; O_3_: ozone; OR: odds ratio; PR: progesterone receptor; PM: particulate matter; PCB: polychlorinated biphenyls; PAH: Polycyclic aromatic hydrocarbon; SNPs: Single Nucleotide Polymorphisms; US: United States

**Acknowledgements**

This work is carried out in partnership with the ARC Foundation for Cancer Research (9 rue Guy Môquet - BP 90003 - 94803 Villejuif Cedex). The E3N cohort is financially supported by Ligue Contre le Cancer, the Mutuelle Générale de l’Education Nationale, the Institut Gustave Roussy, the Institut National de la Santé et de la Recherche. Delphine Praud is supported by a post-doctoral fellowship from the National French Cancer League. Thomas Coudon is granted by a PhD fellowship from University Claude Bernard Lyon 1. The funding body had no role in the conception, design, planning, or writing of the study.

The authors thank all participants for providing data and physicians for providing pathology reports. We thank Camille Denis for data collection and Hassan Hourani for his work on the inventory and characterization of cadmium sources. We thank the scientific committee of the project for its advice on the exposure assessment.

**Ethics approval and consent to participate**

Our research is based on the existing French national cohort E3N. Informed consent was obtained from all participants and the study was approved by the French National Commission for Data Protection and Privacy (CNIL).

**Consent for publication**

All authors have read the manuscript and have agreed to the submission

**Competing interests**

The authors declare they have no actual or potential competing financial interests

**Authors’ contributions**

Conception and study design: BF, JG, and GS. Project management: AA, TC DP, EL, and XM. Supervision: BF, JG, and GS. Exposures assessment/air pollution modelling and geocoding: TC, EF, PS, FC, BB, JC, JG, and, XM. Statistical analyses: AA, DP, KL, EL, and AMND.

Drafting the first version of the manuscript AA, TC, BF. All authors have contributed to writing the manuscript or revising it critically. All authors have read and approved the final manuscript.

**References**

1. Bray F, Ferlay J, Soerjomataram I, Siegel RL, Torre LA, Jemal A. Global cancer statistics 2018: GLOBOCAN estimates of incidence and mortality worldwide for 36 cancers in 185 countries. CA Cancer J Clin [Internet] 2018 [cited 2018 Dec 18];68(6):394–424. [doi: 10.3322/caac.21492]

2. Binder-Foucard F, Bossard N, Delafosse P, Belot A, Woronoff A-S, Remontet L, French network of cancer registries (Francim). Cancer incidence and mortality in France over the 1980-2012 period: solid tumors. Rev Dépidémiologie Santé Publique 2014 Apr;62(2):95–108. [doi: 10.1016/j.respe.2013.11.073]

3. Harvie M, Howell A, Evans DG. Can diet and lifestyle prevent breast cancer: what is the evidence? Am Soc Clin Oncol Educ Book Am Soc Clin Oncol Annu Meet 2015;e66-73. PMID:25993238

4. Dieterich M, Stubert J, Reimer T, Erickson N, Berling A. Influence of lifestyle factors on breast cancer risk. Breast Care Basel Switz 2014 Dec;9(6):407–414. PMID:25759623

5. Jemal A, Center MM, DeSantis C, Ward EM. Global patterns of cancer incidence and mortality rates and trends. Cancer Epidemiol Biomark Prev Publ Am Assoc Cancer Res Cosponsored Am Soc Prev Oncol 2010 Aug;19(8):1893–1907. PMID:20647400

6. Brody JG, Moysich KB, Humblet O, Attfield KR, Beehler GP, Rudel RA. Environmental pollutants and breast cancer: epidemiologic studies. Cancer 2007 Jun 15;109(12 Suppl):2667–2711. PMID:17503436

7. Rudel RA, Ackerman JM, Attfield KR, Brody JG. New exposure biomarkers as tools for breast cancer epidemiology, biomonitoring, and prevention: a systematic approach based on animal evidence. Environ Health Perspect 2014 Sep;122(9):881–895. PMID:24818537

8. WHO | Ambient air pollution [Internet]. WHO. [cited 2018 Aug 24]. Available from: http://www.who.int/airpollution/ambient/en/

9. Loomis D, Grosse Y, Lauby-Secretan B, El Ghissassi F, Bouvard V, Benbrahim-Tallaa L, Guha N, Baan R, Mattock H, Straif K. The carcinogenicity of outdoor air pollution. Lancet Oncol 2013 Dec;14(13):1262–1263. PMID:25035875

10. Wei Y, Davis J, Bina WF. Ambient air pollution is associated with the increased incidence of breast cancer in US. Int J Environ Health Res 2012;22(1):12–21. PMID:21644128

11. Andersen ZJ, Stafoggia M, Weinmayr G, Pedersen M, Galassi C, Jorgensen JT, Oudin A, Forsberg B, Olsson D, Oftedal B, Aasvang GM, Aamodt G, Pyko A, Pershagen G, Korek M, De Faire U, Pedersen NL, Ostenson C-G, Fratiglioni L, Eriksen KT, Tjonneland A, Peeters PH, Bueno-de-Mesquita B, Plusquin M, Key TJ, Jaensch A, Nagel G, Lang A, Wang M, Tsai M-Y, Fournier A, Boutron-Ruault M-C, Baglietto L, Grioni S, Marcon A, Krogh V, Ricceri F, Sacerdote C, Migliore E, Tamayo-Uria I, Amiano P, Dorronsoro M, Vermeulen R, Sokhi R, Keuken M, de Hoogh K, Beelen R, Vineis P, Cesaroni G, Brunekreef B, Hoek G, Raaschou-Nielsen O. Long-Term Exposure to Ambient Air Pollution and Incidence of Postmenopausal Breast Cancer in 15 European Cohorts within the ESCAPE Project. Environ Health Perspect 2017 Oct 13;125(10):107005. PMID:29033383

12. Nie J, Beyea J, Bonner MR, Han D, Vena JE, Rogerson P, Vito D, Muti P, Trevisan M, Edge SB, Freudenheim JL. Exposure to traffic emissions throughout life and risk of breast cancer: the Western New York Exposures and Breast Cancer (WEB) study. Cancer Causes Control CCC 2007 Nov;18(9):947–955. PMID:17632764

13. Raaschou-Nielsen O, Andersen ZJ, Hvidberg M, Jensen SS, Ketzel M, Sorensen M, Hansen J, Loft S, Overvad K, Tjonneland A. Air pollution from traffic and cancer incidence: a Danish cohort study. Environ Health Glob Access Sci Source 2011 Jul 19;10:67. PMID:21771295

14. Hystad P, Villeneuve PJ, Goldberg MS, Crouse DL, Johnson K. Exposure to traffic-related air pollution and the risk of developing breast cancer among women in eight Canadian provinces: a case-control study. Environ Int 2015 Jan;74:240–248. PMID:25454241

15. White AJ, Bradshaw PT, Hamra GB. Air pollution and Breast Cancer: A Review. Curr Epidemiol Rep 2018 Jun;5(2):92–100. PMID:30271702

16. Yaghjyan L, Arao R, Brokamp C, O’Meara ES, Sprague BL, Ghita G, Ryan P. Association between air pollution and mammographic breast density in the Breast Cancer Surveilance Consortium. Breast Cancer Res BCR 2017 Apr 6;19(1):36. PMID:28381271

17. Leng L, Li J, Luo X-M, Kim J-Y, Li Y-M, Guo X-M, Chen X, Yang Q-Y, Li G, Tang N-J. Polychlorinated biphenyls and breast cancer: A congener-specific meta-analysis. Environ Int 2016 Mar;88:133–141. PMID:26735351

18. Lauby-Secretan B, Loomis D, Grosse Y, El Ghissassi F, Bouvard V, Benbrahim-Tallaa L, Guha N, Baan R, Mattock H, Straif K. Carcinogenicity of polychlorinated biphenyls and polybrominated biphenyls. Lancet Oncol 2013 Apr;14(4):287–288. PMID:23499544

19. Dai D, Oyana TJ. Spatial variations in the incidence of breast cancer and potential risks associated with soil dioxin contamination in Midland, Saginaw, and Bay Counties, Michigan, USA. Environ Health Glob Access Sci Source 2008 Oct 21;7:49. PMID:18939976

20. Xu J, Ye Y, Huang F, Chen H, Wu H, Huang J, Hu J, Xia D, Wu Y. Association between dioxin and cancer incidence and mortality: a meta-analysis. Sci Rep [Internet] 2016 Dec [cited 2017 Aug 3];6(1). [doi: 10.1038/srep38012]

21. Rodgers KM, Udesky JO, Rudel RA, Brody JG. Environmental chemicals and breast cancer: An updated review of epidemiological literature informed by biological mechanisms. Environ Res 2018 Jan;160:152–182. PMID:28987728

22. Salehi F, Turner MC, Phillips KP, Wigle DT, Krewski D, Aronson KJ. Review of the etiology of breast cancer with special attention to organochlorines as potential endocrine disruptors. J Toxicol Environ Health B Crit Rev 2008 Mar;11(3–4):276–300. PMID:18368557

23. Liu R, Nelson DO, Hurley S, Hertz A, Reynolds P. Residential exposure to estrogen disrupting hazardous air pollutants and breast cancer risk: the California Teachers Study. Epidemiol Camb Mass 2015 May;26(3):365–373. PMID:25760782

24. Julin B, Wolk A, Bergkvist L, Bottai M, Akesson A. Dietary cadmium exposure and risk of postmenopausal breast cancer: a population-based prospective cohort study. Cancer Res 2012 Mar 15;72(6):1459–1466. PMID:22422990

25. Mordukhovich I, Beyea J, Herring AH, Hatch M, Stellman SD, Teitelbaum SL, Richardson DB, Millikan RC, Engel LS, Shantakumar S, Steck SE, Neugut AI, Rossner P, Santella RM, Gammon MD. Vehicular Traffic-Related Polycyclic Aromatic Hydrocarbon Exposure and Breast Cancer Incidence: The Long Island Breast Cancer Study Project (LIBCSP). Environ Health Perspect 2016 Jan;124(1):30–38. PMID:26008800

26. Bonner MR, Han D, Nie J, Rogerson P, Vena JE, Muti P, Trevisan M, Edge SB, Freudenheim JL. Breast cancer risk and exposure in early life to polycyclic aromatic hydrocarbons using total suspended particulates as a proxy measure. Cancer Epidemiol Biomark Prev Publ Am Assoc Cancer Res Cosponsored Am Soc Prev Oncol 2005 Jan;14(1):53–60. PMID:15668476

27. Straif K, Benbrahim-Tallaa L, Baan R, Grosse Y, Secretan B, El Ghissassi F, Bouvard V, Guha N, Freeman C, Galichet L, Cogliano V. A review of human carcinogens--Part C: metals, arsenic, dusts, and fibres. Lancet Oncol 2009 May;10(5):453–454. PMID:19418618

28. Liu R, Nelson DO, Hurley S, Hertz A, Reynolds P. Residential exposure to estrogen disrupting hazardous air pollutants and breast cancer risk: the California Teachers Study. Epidemiol Camb Mass 2015 May;26(3):365–373. PMID:25760782

29. WHO | Household air pollution [Internet]. WHO. [cited 2018 Aug 24]. Available from: http://www.who.int/airpollution/household/en/

30. Bruce N, Dherani M, Liu R, Hosgood HD 3rd, Sapkota A, Smith KR, Straif K, Lan Q, Pope D. Does household use of biomass fuel cause lung cancer? A systematic review and evaluation of the evidence for the GBD 2010 study. Thorax 2015 May;70(5):433–441. PMID:25758120

31. Global, regional, and national comparative risk assessment of 79 behavioural, environmental and occupational, and metabolic risks or clusters of risks,. Lancet Lond Engl 2016 Oct 8;388(10053):1659–1724. PMID:27733284

32. Yuan Y, Luo Z, Liu J, Wang Y, Lin Y. Health and economic benefits of building ventilation interventions for reducing indoor PM2.5 exposure from both indoor and outdoor origins in urban Beijing, China. Sci Total Environ 2018 Jun 1;626:546–554. PMID:29353793

33. Villanueva F, Tapia A, Lara S, Amo-Salas M. Indoor and outdoor air concentrations of volatile organic compounds and NO2 in schools of urban, industrial and rural areas in Central-Southern Spain. Sci Total Environ 2018 May 1;622–623:222–235. PMID:29212055

34. Stabile L, Buonanno G, Avino P, Frattolillo A, Guerriero E. Indoor exposure to particles emitted by biomass-burning heating systems and evaluation of dose and lung cancer risk received by population. Environ Pollut Barking Essex 1987 2018 Apr;235:65–73. PMID:29274539

35. Jorquera H, Barraza F, Heyer J, Valdivia G, Schiappacasse LN, Montoya LD. Indoor PM2.5 in an urban zone with heavy wood smoke pollution: The case of Temuco, Chile. Environ Pollut Barking Essex 1987 2018 May;236:477–487. PMID:29414372

36. White AJ, Teitelbaum SL, Stellman SD, Beyea J, Steck SE, Mordukhovich I, McCarty KM, Ahn J, Rossner PJ, Santella RM, Gammon MD. Indoor air pollution exposure from use of indoor stoves and fireplaces in association with breast cancer: a case-control study. Environ Health Glob Access Sci Source 2014 Dec 12;13:108. PMID:25495350

37. White AJ, Sandler DP. Indoor Wood-Burning Stove and Fireplace Use and Breast Cancer in a Prospective Cohort Study. Environ Health Perspect 2017 Jul 18;125(7):077011. PMID:28728136

38. Perou CM, Sorlie T, Eisen MB, van de Rijn M, Jeffrey SS, Rees CA, Pollack JR, Ross DT, Johnsen H, Akslen LA, Fluge O, Pergamenschikov A, Williams C, Zhu SX, Lonning PE, Borresen-Dale AL, Brown PO, Botstein D. Molecular portraits of human breast tumours. Nature 2000 Aug 17;406(6797):747–752. PMID:10963602

39. Ogino S, Nishihara R, VanderWeele TJ, Wang M, Nishi A, Lochhead P, Qian ZR, Zhang X, Wu K, Nan H, Yoshida K, Milner DAJ, Chan AT, Field AE, Camargo CAJ, Williams MA, Giovannucci EL. Review Article: The Role of Molecular Pathological Epidemiology in the Study of Neoplastic and Non-neoplastic Diseases in the Era of Precision Medicine. Epidemiol Camb Mass 2016 Jul;27(4):602–611. PMID:26928707

40. Ogino S, Nowak JA, Hamada T, Milner DAJ, Nishihara R. Insights into Pathogenic Interactions Among Environment, Host, and Tumor at the Crossroads of Molecular Pathology and Epidemiology. Annu Rev Pathol 2019 Jan 24;14:83–103. PMID:30125150

41. Basagaña X, Aguilera I, Rivera M, Agis D, Foraster M, Marrugat J, Elosua R, Künzli N. Measurement error in epidemiologic studies of air pollution based on land-use regression models. Am J Epidemiol 2013 Oct 15;178(8):1342–1346. PMID:24105967

42. Wilton D, Szpiro A, Gould T, Larson T. Improving spatial concentration estimates for nitrogen oxides using a hybrid meteorological dispersion/land use regression model in Los Angeles, CA and Seattle, WA. Sci Total Environ [Internet] 2010 Feb [cited 2017 Aug 21];408(5):1120–1130. [doi: 10.1016/j.scitotenv.2009.11.033]

43. Coudon T, Hourani H, Nguyen C, Faure E, Mancini FR, Fervers B, Salizzoni P. Assessment of long-term exposure to airborne dioxin and cadmium concentrations in the Lyon metropolitan area (France). Environ Int 2018 Feb;111:177–190. PMID:29220728

44. Potischman N, Troisi R. In-utero and early life exposures in relation to risk of breast cancer. Cancer Causes Control CCC 1999 Dec;10(6):561–573. PMID:10616825

45. Cohn BA, Wolff MS, Cirillo PM, Sholtz RI. DDT and breast cancer in young women: new data on the significance of age at exposure. Environ Health Perspect 2007 Oct;115(10):1406–1414. PMID:17938728

46. Beyea J, Stellman SD, Teitelbaum S, Mordukhovich I, Gammon MD. Imputation method for lifetime exposure assessment in air pollution epidemiologic studies. Environ Health Glob Access Sci Source 2013 Aug 7;12:62. PMID:23919666

47. Binachon B, Dossus L, Danjou AMN, Clavel-Chapelon F, Fervers B. Life in urban areas and breast cancer risk in the French E3N cohort. Eur J Epidemiol 2014 Oct;29(10):743–751. PMID:25139141

48. Lambrechtsen J, Gerke O, Egstrup K, Sand NP, Norgaard BL, Petersen H, Mickley H, Diederichsen ACP. The relation between coronary artery calcification in asymptomatic subjects and both traditional risk factors and living in the city centre: a DanRisk substudy. J Intern Med 2012 May;271(5):444–450. PMID:22092933

49. Wei T, Jia J, Wada Y, Kapron CM, Liu J. Dose dependent effects of cadmium on tumor angiogenesis. Oncotarget 2017 Jul 4;8(27):44944–44959. PMID:28388546

50. Wild CP. Complementing the genome with an “exposome”: the outstanding challenge of environmental exposure measurement in molecular epidemiology. Cancer Epidemiol Biomark Prev Publ Am Assoc Cancer Res Cosponsored Am Soc Prev Oncol 2005 Aug;14(8):1847–1850. PMID:16103423

51. Billionnet C, Sherrill D, Annesi-Maesano I. Estimating the health effects of exposure to multi-pollutant mixture. Ann Epidemiol 2012 Feb;22(2):126–141. PMID:22226033

52. Oakes M, Baxter L, Long TC. Evaluating the application of multipollutant exposure metrics in air pollution health studies. Environ Int 2014 Aug;69:90–99. PMID:24815342

53. Go R-E, Hwang K-A, Kim C-W, Byun Y-S, Nam K-H, Choi K-C. Effect of dioxin and 17beta-estradiol on the expression of cytochrome P450 1A1 gene via an estrogen receptor dependent pathway in cellular and xenografted models. Environ Toxicol 2017 Oct;32(10):2225–2233. PMID:28618207

54. Yang B, Fan S, Zhi X, Xia R, Wang Y, Zheng Q, Sun G. Geographical and ethnic distribution of MTHFR gene polymorphisms and their associations with diseases among Chinese population. Clin Genet 2017 Sep;92(3):243–258. PMID:27888505

55. Saintot M, Malaveille C, Hautefeuille A, Gerber M. Interaction between genetic polymorphism of cytochrome P450-1B1 and environmental pollutants in breast cancer risk. Eur J Cancer Prev Off J Eur Cancer Prev Organ ECP 2004 Feb;13(1):83–86. PMID:15075793

56. Laden F, Ishibe N, Hankinson SE, Wolff MS, Gertig DM, Hunter DJ, Kelsey KT. Polychlorinated biphenyls, cytochrome P450 1A1, and breast cancer risk in the Nurses’ Health Study. Cancer Epidemiol Biomark Prev Publ Am Assoc Cancer Res Cosponsored Am Soc Prev Oncol 2002 Dec;11(12):1560–1565. PMID:12496044

57. Cortessis VK, Thomas DC, Levine AJ, Breton CV, Mack TM, Siegmund KD, Haile RW, Laird PW. Environmental epigenetics: prospects for studying epigenetic mediation of exposure-response relationships. Hum Genet 2012 Oct;131(10):1565–1589. PMID:22740325

58. Hou L, Zhang X, Wang D, Baccarelli A. Environmental chemical exposures and human epigenetics. Int J Epidemiol 2012 Feb;41(1):79–105. PMID:22253299

59. Demetriou CA, Vineis P. Carcinogenicity of ambient air pollution: use of biomarkers, lessons learnt and future directions. J Thorac Dis 2015 Jan;7(1):67–95. PMID:25694819

60. Peluso M, Bollati V, Munnia A, Srivatanakul P, Jedpiyawongse A, Sangrajrang S, Piro S, Ceppi M, Bertazzi PA, Boffetta P, Baccarelli AA. DNA methylation differences in exposed workers and nearby residents of the Ma Ta Phut industrial estate, Rayong, Thailand. Int J Epidemiol 2012 Dec;41(6):1753–60; discussion 1761-1763. PMID:23064502

61. Demetriou CA, Raaschou-Nielsen O, Loft S, Moller P, Vermeulen R, Palli D, Chadeau-Hyam M, Xun WW, Vineis P. Biomarkers of ambient air pollution and lung cancer: a systematic review. Occup Environ Med 2012 Sep;69(9):619–627. PMID:22773658

62. Clavel-Chapelon F. Cohort Profile: The French E3N Cohort Study. Int J Epidemiol 2015 Jun;44(3):801–809. PMID:25212479

63. Riboli E, Hunt KJ, Slimani N, Ferrari P, Norat T, Fahey M, Charrondière UR, Hémon B, Casagrande C, Vignat J, Overvad K, Tjønneland A, Clavel-Chapelon F, Thiébaut A, Wahrendorf J, Boeing H, Trichopoulos D, Trichopoulou A, Vineis P, Palli D, Bueno-De-Mesquita HB, Peeters PHM, Lund E, Engeset D, González CA, Barricarte A, Berglund G, Hallmans G, Day NE, Key TJ, Kaaks R, Saracci R. European Prospective Investigation into Cancer and Nutrition (EPIC): study populations and data collection. Public Health Nutr 2002 Dec;5(6B):1113–1124. PMID:12639222

64. Binachon B, Dossus L, Danjou AMN, Clavel-Chapelon F, Fervers B. Life in urban areas and breast cancer risk in the French E3N cohort. Eur J Epidemiol 2014 Oct;29(10):743–751. [doi: 10.1007/s10654-014-9942-z]

65. Lucas F, Niravong M, Villeminot S, Kaaks R, Clavel‐Chapelon F. Estimation of food portion size using photographs: validity, strengths, weaknesses and recommendations. J Hum Nutr Diet [Internet] 1995 Feb 1 [cited 2018 Nov 21];8(1):65–74. [doi: 10.1111/j.1365-277X.1995.tb00296.x]

66. van Liere MJ, Lucas F, Clavel F, Slimani N, Villeminot S. Relative validity and reproducibility of a French dietary history questionnaire. Int J Epidemiol 1997;26 Suppl 1:S128-136. PMID:9126541

67. Leblanc J-C, Guérin T, Noël L, Calamassi-Tran G, Volatier J-L, Verger P. Dietary exposure estimates of 18 elements from the 1st French Total Diet Study. Food Addit Contam 2005 Jul;22(7):624–641. PMID:16019838

68. Sirot V, Tard A, Venisseau A, Brosseaud A, Marchand P, Le Bizec B, Leblanc J-C. Dietary exposure to polychlorinated dibenzo-p-dioxins, polychlorinated dibenzofurans and polychlorinated biphenyls of the French population: Results of the second French Total Diet Study. Chemosphere 2012 Jul;88(4):492–500. PMID:22487562

69. Faure E, Danjou AMN, Clavel-Chapelon F, Boutron-Ruault M-C, Dossus L, Fervers B. Accuracy of two geocoding methods for geographic information system-based exposure assessment in epidemiological studies. Environ Health Glob Access Sci Source 2017 Feb 24;16(1):15. PMID:28235407

70. Couvidat F, Bessagnet B, Garcia-Vivanco M, Real E, Menut L, Colette A. Development of an inorganic and organic aerosol model (CHIMERE 2017*β* v1.0): seasonal and spatial evaluation over Europe. Geosci Model Dev [Internet] 2018 Jan 15 [cited 2019 Feb 28];11(1):165–194. [doi: https://doi.org/10.5194/gmd-11-165-2018]

71. Soulhac L, Nguyen C, Volta P, Salizzoni P. The model SIRANE for atmospheric urban pollutant dispersion. PART III: Validation against NO2 yearly concentration measurements in a large urban agglomeration. Atmos Env 2017;167:377–388. [doi: doi:10.1016/j.atmosenv.2017.08.034]

72. Gulliver J, de Hoogh K, Hansell A, Vienneau D. Development and Back-Extrapolation of NO _2_ Land Use Regression Models for Historic Exposure Assessment in Great Britain. Environ Sci Technol [Internet] 2013 Jul 16 [cited 2017 Aug 22];47(14):7804–7811. [doi: 10.1021/es4008849]

73. Coudon T, Danjou AMN, Faure E, Praud D, Severi G, Mancini FR, Salizzoni P, Fervers B. Development and performance evaluation of a GIS-based metric to assess exposure to airborne pollutant emissions from industrial sources. Environ Health Glob Access Sci Source 2019 25;18(1):8. PMID:30683108

74. Danjou AMN, Coudon T, Praud D, Lévêque E, Faure E, Salizzoni P, Le Romancer M, Severi G, Mancini FR, Leffondré K, Dossus L, Fervers B. Long-term airborne dioxin exposure and breast cancer risk in a case-control study nested within the French E3N prospective cohort. Environ Int 2019 Mar;124:236–248. PMID:30658268

75. Guerreiro CBB, Horálek J, de Leeuw F, Couvidat F. Benzo(a)pyrene in Europe: Ambient air concentrations, population exposure and health effects. Environ Pollut Barking Essex 1987 2016 Jul;214:657–667. PMID:27140679

76. Wang M, Spiegelman D, Kuchiba A, Lochhead P, Kim S, Chan AT, Poole EM, Tamimi R, Tworoger SS, Giovannucci E, Rosner B, Ogino S. Statistical methods for studying disease subtype heterogeneity. Stat Med 2016 Feb 28;35(5):782–800. PMID:26619806

77. Durrleman S, Simon R. Flexible regression models with cubic splines. Stat Med 1989 May;8(5):551–561. PMID:2657958

78. Desquilbet L, Mariotti F. Dose-response analyses using restricted cubic spline functions in public health research. Stat Med 2010 Apr 30;29(9):1037–1057. PMID:20087875

79. Lacourt A, Leffondre K, Gramond C, Ducamp S, Rolland P, Gilg Soit Ilg A, Houot M, Imbernon E, Fevotte J, Goldberg M, Brochard P. Temporal patterns of occupational asbestos exposure and risk of pleural mesothelioma. Eur Respir J 2012 Jun;39(6):1304–1312. PMID:22075480

80. Lévêque E, Lacourt A, Luce D, Sylvestre M-P, Guénel P, Stücker I, Leffondré K. Time-dependent effect of intensity of smoking and of occupational exposure to asbestos on the risk of lung cancer: results from the ICARE case-control study. Occup Environ Med 2018 Aug;75(8):586–592. PMID:29777039

81. Lacourt A, Lévêque E, Guichard E, Gilg Soit Ilg A, Sylvestre M-P, Leffondré K. Dose-time-response association between occupational asbestos exposure and pleural mesothelioma. Occup Environ Med 2017;74(9):691–697. PMID:28501798

82. McCulloch CE, Lin H, Slate EH, Turnbull BW. Discovering subpopulation structure with latent class mixed models. Stat Med 2002 Feb 15;21(3):417–429. PMID:11813228

83. Stafoggia M, Breitner S, Hampel R, Basagana X. Statistical Approaches to Address Multi-Pollutant Mixtures and Multiple Exposures: the State of the Science. Curr Environ Health Rep 2017 Dec;4(4):481–490. PMID:28988291

84. Dupont WD, Plummer WDJ. Power and sample size calculations. A review and computer program. Control Clin Trials 1990 Apr;11(2):116–128. PMID:2161310

85. Hutchings S. The burden of occupational cancer in Great Britain. :68.

86. Benjamin L, Cotte F-E, Mercier F, Vainchtock A, Vidal-Trecan G, Durand-Zaleski I. Burden of breast cancer with brain metastasis: a French national hospital database analysis. J Med Econ 2012;15(3):493–499. PMID:22304337

87. Jay N, Nuemi G, Gadreau M, Quantin C. A data mining approach for grouping and analyzing trajectories of care using claim data: the example of breast cancer. BMC Med Inform Decis Mak 2013 Nov 30;13:130. PMID:24289668

88. Danjou AMN, Coudon T, Praud D, Lévêque E, Faure E, Salizzoni P, Le Romancer M, Severi G, Mancini FR, Leffondré K, Dossus L, Fervers B. Long-term Airborne Dioxin Exposure and Breast Cancer Risk in a case-control study nested within the French E3N Prospective Cohort. Press Environ Int 2019;

89. Coudon T, Salizzoni P, Praud D, Danjou A, Dossus L, Faure E. National inventory of historical dioxin air emission sources in France. Press Atmospheric Pollut Res 2018;

90. de Hoogh K, Gulliver J, Donkelaar A van, Martin RV, Marshall JD, Bechle MJ, Cesaroni G, Pradas MC, Dedele A, Eeftens M, Forsberg B, Galassi C, Heinrich J, Hoffmann B, Jacquemin B, Katsouyanni K, Korek M, Künzli N, Lindley SJ, Lepeule J, Meleux F, de Nazelle A, Nieuwenhuijsen M, Nystad W, Raaschou-Nielsen O, Peters A, Peuch V-H, Rouil L, Udvardy O, Slama R, Stempfelet M, Stephanou EG, Tsai MY, Yli-Tuomi T, Weinmayr G, Brunekreef B, Vienneau D, Hoek G. Development of West-European PM 2.5 and NO 2 land use regression models incorporating satellite-derived and chemical transport modelling data. Environ Res [Internet] 2016 Nov [cited 2017 Aug 3];151:1–10. [doi: 10.1016/j.envres.2016.07.005]
